# Systematic Development of Vanadium Catalysts for Sustainable Epoxidation of Small Alkenes and Allylic Alcohols

**DOI:** 10.3390/ijms241512299

**Published:** 2023-08-01

**Authors:** José Ferraz-Caetano, Filipe Teixeira, Maria Natália Dias Soeiro Cordeiro

**Affiliations:** 1 LAQV-REQUIMTE, Department of Chemistry and Biochemistry, Faculty of Sciences, University of Porto, Rua do Campo Alegre, S/N, 4169-007 Porto, Portugal; jose.caetano@fc.up.pt; 2 CQUM, Centre of Chemistry, University of Minho, Campus de Gualtar, 4710-057 Braga, Portugal; fteixeira@quimica.uminho.pt

**Keywords:** alkene epoxidation, allylic alcohol epoxidation, vanadium catalysts, sustainable chemistry

## Abstract

The catalytic epoxidation of small alkenes and allylic alcohols includes a wide range of valuable chemical applications, with many works describing vanadium complexes as suitable catalysts towards sustainable process chemistry. But, given the complexity of these mechanisms, it is not always easy to sort out efficient examples for streamlining sustainable processes and tuning product optimization. In this review, we provide an update on major works of tunable vanadium-catalyzed epoxidations, with a focus on sustainable optimization routes. After presenting the current mechanistic view on vanadium catalysts for small alkenes and allylic alcohols’ epoxidation, we argue the key challenges in green process development by highlighting the value of updated kinetic and mechanistic studies, along with essential computational studies.

## 1. Introduction

**Figure 1:**
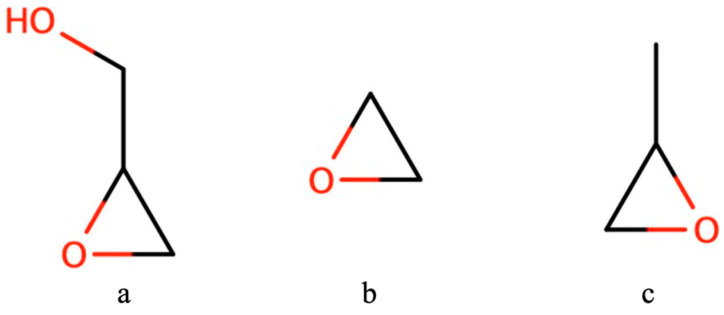
Common commercial epoxides (from left to right): glycidol (oxiran-2-yl)methanol)—(**a**); ethylene oxide (oxirane)—(**b**); propylene oxide (2-methyloxirane)—(**c**).

Epoxides are cyclical chemical structures displaying a triangular arrangement of two carbon atoms and one oxygen (Figure 1) [1,2,3]. These apparently simple structures are considerably reactive, harnessing great chemical energy from their equilateral atom arrangement. More specifically, epoxides are highly reactive to ring opening as the stress caused by the valence angles (60°) is much lower than the expected angle for atoms with sp^3^ hybridization (109°). They are also pivotal intermediates in the synthesis of complex molecules, given their lability from the often-perceived opening of their two carbon atoms in the epoxidation process [4].

**Figure 2:**
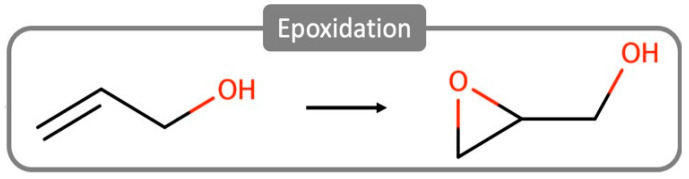
Schematic representation of allylic alcohol epoxidation.

The epoxidation of small alkenes and allylic alcohols (ESAs) is an important synthetic route in the chemical industry (Figure 2) [5,6]. This reaction is one of the building blocks of biologically active molecules and fine pharmaceuticals, prominently used to manufacture valuable commodities. For example, ethylene-oxide (the simplest epoxide) represents a roughly USD 20 billion industry, partially explained because of the extraction costs of petroleum-based epoxide sources [7]. It is, therefore, essential to find alternative sources of raw materials, providing environmental and economic sustainability to epoxide production.

Some of the challenges of ESAs had already been addressed by industrialists, such as feedstock consumption and capital/energy expenditure, while reaction parameter developments were based on designing novel catalysts [8,9]. Despite proven optimized key reaction features, production costs and carbon emissions are still high. To reduce feedstock consumption, biomass-based production routes of ESAs are growing momentum [9,10]. Promising accessible bio-sources include lipids (vegetable oils [11,12]), terpenes (fruit and tree wastes [13]) and lignocellulosic compounds (plant husks [14]), vowing to reduce the industry’s carbon footprint. Notwithstanding encouraging results for reaction yield and selectivity, developments towards industrial scale-up applications are still pending.

**Figure 3:**
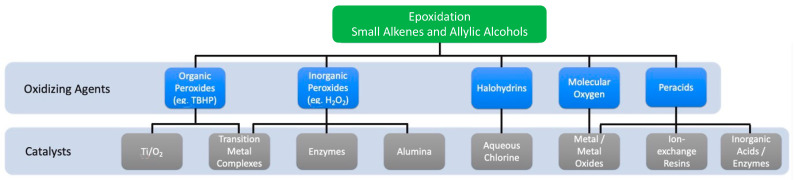
Graphical distribution of the possible epoxidation routes for small alkenes and allylic alcohol by oxidizing agents and catalysts.

Historically, several tentative new routes have been reported considering epoxide complexity. Early developments emerge from the epoxidation of small alkenes through a chemical base, bearing the reaction with molecular oxygen and using silver as a catalyst (Wurtz’s method [15]). Efforts then turned to the synthesis of complex epoxides using different oxidants, namely peroxyacids [16,17], oxones [18,19] and dioxiranes [20,21]. But resource concerns on the sustainability of these processes deemed them costly (in particular with molecular oxygen), with increasingly high by-product turnouts. To overcome environmental and production concerns, the catalytic developments for epoxidation reactions have been thoroughly researched. An interesting take on the categorization of catalytic epoxidation reactions, featuring small alkenes and allylic alcohols, was recently presented by Meng [22]. As we can see in Figure 3, epoxidation reactions can be sorted by oxidizing agents and the nature of their catalyst.

Considering the trade-off in the sustainable shift of oxidant/catalyst pairs, complex ESAs’ reactions are often accompanied by the use of transition-metal complexes as reaction catalysts. The most sought-out structures (in particular with industrial applications) are manganese–salen complexes [23], metalloporphyrins [24,25,26] and other transition metal complexes containing titanium, molybdenum or vanadium [27,28,29,30]. Indeed, solid-phase industrial epoxidation catalytic processes are extensively reported in the literature [31,32,33]. The development of chiral epoxide synthesis from small-chain substrates is based on the original concept of Katsuki and Sharpless [27] using Mn(salen) derivatives, or with other transition metals such as vanadium [34], Mn(porphyrin) [35] and Fe(porphyrin) [36]. Despite their efficiency, these methods present performance issues due to catalyst loading and require an anhydrous reaction environment [37]. Therefore, there is still a long way to go in improving catalytic efficiency, given the cost of these materials and low recovery turnouts, focusing the development on circular economy-friendly strategies. One popular strategy concerns metalorganic frameworks (MOFs) as an interesting catalyst encapsuling playgrounds for the development of heterogeneous catalytic epoxidation vanadium systems [38,39,40]. Despite their promising results in catalyst selectivity, there are still limitations regarding industrial large-scale applications. Since we opted to highlight simple, cost-effective mediums for potential epoxidation industrial applications, we refer the reader to a review on MOF uses for vanadium-based epoxidation [40].

To highlight sustainable developments in catalytic ESAs processes, this review takes into consideration benchmark routes using vanadium complexes as catalysts. Vanadium was chosen due to its wide application in chemical catalysis, backed by many articles published in recent years. Considering the data provided in Table 1, there have been approximately 25 review articles on epoxidation with vanadium catalysts since the early 1990s. Out of them, we selected the important works of Buhl [41] (2004), Gupta [42] (2008), Sutradhar [43] (2015) and Irshad [10] (2022), concerning the state-of-the-art of epoxidation of small organic molecules, as other reviews emphasized substrates with larger and/or polymeric substrates [44].

The goal of this work is to categorize fundamental works in the development of sustainable ESAs with cost-effective vanadium catalysts, as an effort to streamline the search for efficient research databases. To identify possible reaction variables towards eco-friendly processes, we attempt to advance a compendium for chemical experimentalists and theoreticians regarding experimental practices and developments with vanadium-based epoxidation reactions. Due to the increasing demand for short-chain raw materials for their favorable environmental and health characteristics, we have limited this review’s key substrates to small alcohols and allylic acids. The major criteria for selecting research studies were the description of low-cost reaction mediums (with less organic solvents), cost-effective oxidants and non-complex catalytic structures (usually through a vanadium-based scaffold). We will start by reviewing the development of vanadium as an epoxidation catalyst, showcasing new progress in epoxidation mechanisms. With emphasis on two major substrates (allylic alcohols and small akenes), this article highlights diverse combination strategies of oxidizing agents, substrates and reaction conditions with a particular vanadium-based catalyst.

## 2. Epoxidation of Allylic Alcohols and Small Alkenes Using Vanadium Catalysts

### 2.1. Contextualization

**Figure 4:**
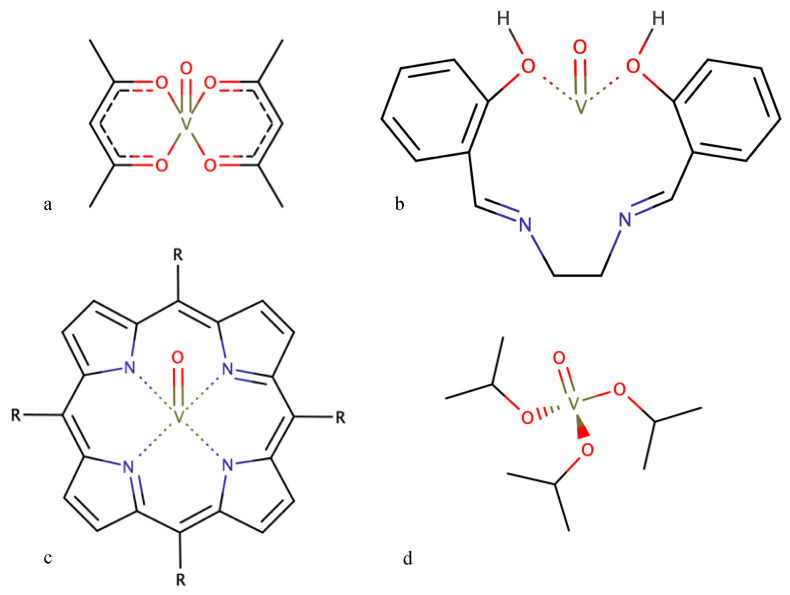
Some examples of vanadium-based catalyst complexes [VO(acac)_2_] (**a**); vanadium(IV) complexes with Schiff base ligands (**b**); VO(porphyrin)-complexes (**c**); and [VO(O*i*Pr)_3_] (**d**).

Vanadium-based chemical catalysis has been thoroughly used for epoxidation of several small-chain substrates, namely alkenes, olefins and allylic alcohols [31]. As pointed out by Langeslay in 2019, this is due to regioselective or stereoselective epoxidation yielding chiral epoxides [45]. Given their ability to transfer oxygen atoms onto the substrate, vanadium-based catalysts are predominantly chosen because of their selectivity on a preparative scale. Four popular structures can be named in industry-wide applications. First, epoxidation with vanadyl acetylacetonate-based complexes—[VO(acac)_2_]—are widely understood as the most popular structures. They go back to the published works of Sharpless in the 1970s [27], as this framework spun novel purposes for this process. These complexes have been described for alkene and allylic alcohol epoxidation (Figure 4a) with revamped catalytic activity, considering the lack of ligand electron–donor substituents [46]. The following works focused on another catalyst structure with increasing complexity, both in liquid and solid systems: oxo-vanadium(IV) complexes with Schiff base ligands (Figure 4b) [47,48]. These tetradentate structures were highlighted by Mohebbi in 2006 where substituted vanadium(IV) complexes with N- or O-donor ligands could conduct the catalytic epoxidation of olefins with interesting overall yields. Also based on these developments, Alvarez [49] reported a new structure of oxo-vanadium complexes. The vanadyl acetylacetonate-salen—[VO(salen)]—type structures were able to catalyze the epoxidation of small alkenes using hydrogen peroxide as the oxidant, while also yielding a significant amount of a ketone as a by-product [49,50]. With the relative success of [VO(salen)] catalysts, we can also find mentions of VO(porphyrin)-complexes (Figure 4c), which are widely studied in further applications [51,52,53,54]. And finally, a more recent take has reported the use of vanadyl isopropoxide [55]—[VO(O*i*Pr)_3_]—a common alkoxide of vanadium (Figure 4d), in catalyst systems with noteworthy features such as low catalyst loading, at mild reaction conditions, for the enantioselective desymmetrization of allylic alcohols.

As recently stated by Langeslay, research development in these catalytic systems focused on improving stereoselectivity, optimizing reaction settings and reducing catalyst loading [45]. But perhaps the main concern is the deceleration effect which reduces catalytic efficiency. This was mentioned by Pellissier [37] back in 2015, despite some breakthroughs by operating ligand quantities in reaction mediums. In fact, the trade-off between ligand excess and reaction activity is still critical among its various iterations, as first highlighted by Kirihara and Conte in 2011 [56,57].

Another reason to argue for the use of vanadium are its oxidation states. This is relevant when comparing it with other metals like iron or manganese, since they have more oxidation states beyond other negative features. For example, the reduction potential from V(V) to V(IV), where vanadium complexes are often used in several catalytic reactions of industrial interest such as haloperoxidations and sulfoxidations [56].

### 2.2. Epoxidation Mechanism Overview

**Figure 5:**
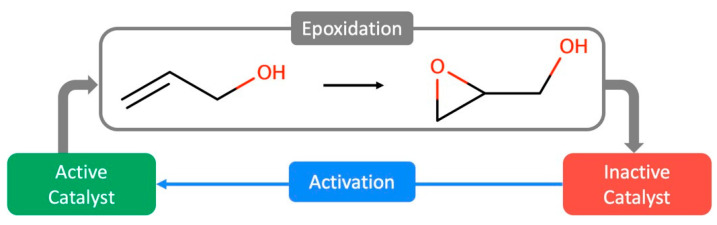
Schematic overview of the oxovanadium catalytic system for the epoxidation of olefins and allylic alcohols. Side reactions represent the inception of active complexes.

The catalytic epoxidation of small-chain alkenes, olefins and allylic alcohols into their corresponding epoxides is a reaction pathway with plenty of applications (Figure 5). It is broadly used in making high-value intermediates of fine pharmaceuticals, plastics, dyes, solvents, flavors and fragrances. The emergence of the reaction’s active complex occurs in the presence of the reaction oxidant, as described in many experimental routes [58,59].

**Figure 6:**
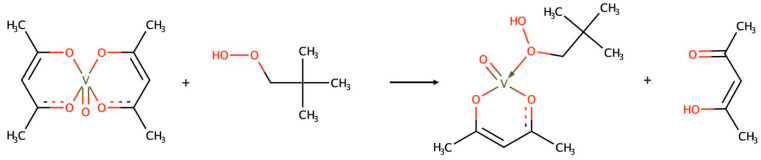
Proposed mechanism for the irreversible formation of an active epoxidation catalyst from [VO(acac)_2_] complex in the presence of acetic acid.

Given the difficulties in acquiring robust mechanistic data for a broader theory on this type of catalytic epoxidation, the literature converges on three different mechanisms of vanadium-based epoxidation catalysts using an oxidizing agent [57]. Nevertheless, for each of these processes to occur, there is a compulsory step in which the catalyst is converted into its active form. Out of the many examples for catalyst activation, Figure 6 depicts a suggested mechanism for a vanadium catalyst, [VO(acac)_2_]. This process is irreversible because of the oxidation of the last acetylacetonate ligand to acetic acid, as it becomes less abundant as the epoxidation develops. The process develops in a two-step method (Figure 5): (i) the epoxidation reaction which converts the catalyst into its inactive form; and (ii) the transition of inactive to active complexes, due to an oxidizing agent.

As explained by Vandichel in 2012 [58], there are other triggering stages besides the initial activation step. Figure 6 shows a non-restrictive example, as vanadium (IV) and vanadium (V) mono-peroxo-complexes can be formed by various ligand exchange reactions. Despite the acetylacetone–(acac)–ligand being the most common choice, other ligands ranging from hydroxyl to acetate (or even tert-butoxide anion) can be formed. Kirillova [60] proposed that these ligand exchange reactions share their origin by bearing other active complexes as equilibrium steps for changing their oxidation state.

Concerning the reaction’s oxidizing agents, the literature on ESAs with vanadium-based complexes highlights molecular oxygen (O_2_), hydrogen peroxide (H_2_O_2_) or organic peroxides as common oxidants. This choice is based on kinetic/spectroscopy studies and theoretical data which corroborate experimental results. In the course of this review, focus will be given to various epoxidation reactions using different oxidants, highlighting the three main pathways for epoxidation involving electrophilic oxygen transfers for vanadium-based catalysts [45]. Given the several alternatives for developing the substrate or ligand’s subsequent peroxo-complex nature, the determining action in these pathways is the vanadium oxidation leading to the complex’s formation.

**Figure 7:**
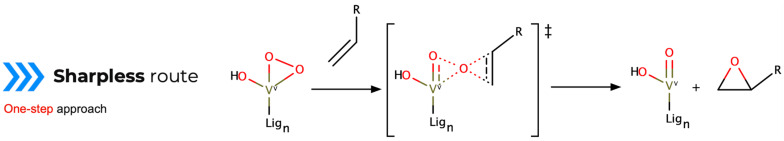
Mechanism outline for epoxidation Sharpless route (‡ = transition state) [34].

The first pathway is the Sharpless route (Figure 7). It is a non-radical mechanism that occurs in a one-step approach with addition of the peroxoxygen, preceded by the nucleophilic attack by the olefin, finishing off with the regeneration of the initial catalyst [34].

**Figure 8:**
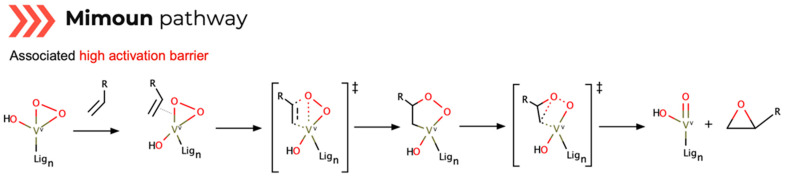
Mechanism outline for epoxidation Mimoun-type route (‡ = transition state) [61].

The second, the Mimoun pathway (Figure 8), is a non-radical mechanism where the substrate is coordinated into the metallic center. The vanadium-oxygen bond forms an intermediate that rules out the produced epoxide while regenerating the catalyst. Despite presenting a straightforward mechanism, its first step’s high reaction activation energy is deemed prohibitive, as backed by several computational calculations [61].

**Figure 9:**
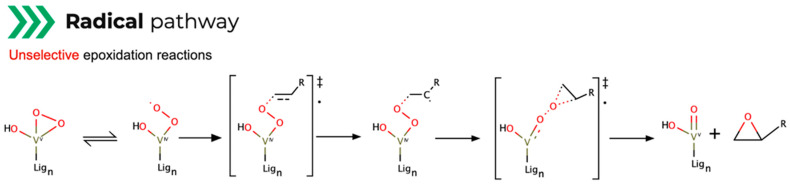
Mechanism outline for epoxidation Radical pathway route (‡ = transition state; **·** = radical) [57].

The third route, the radical pathway (Figure 9), generates a superoxol-complex through a biradical alkylperoxo intermediate, rearranged to regenerate the catalyst while forming the epoxide [57]. The pivotal step is the formation of a radical cation–anion pair (allowing a competitive radical process to occur), as this pathway performs unselective epoxidation reactions [57]. This is due to the nature of the mechanism involving a homolytic rupture of one metal–peroxo oxygen, downplaying its catalytic efficiency.

Out of the three mechanisms, Mimoun is seldom cited because of its associated high activation barrier. However, research efforts endure on the clarification of structure and reactivity correlation in these routes. For each mechanism, there are two commonly quoted vanadium-based catalyst ligand scaffolds for these purposes: salen and vanadyl-based [45].

### 2.3. Comparative Theoretical Studies

To evaluate the preferred reaction route, comparative theoretical studies on the three epoxidation mechanisms with vanadium-based catalysts in the presence of an oxidizing agent were performed [58]. Using DFT level of theory through the optimization of transition structures, combined with correlation functional Becke’s three-parameter hybrid exchange functional with Lee, Yang and Parr gradient-corrected correlation functional (B3LYP), Vandichel carried out single-point calculations using the 6-311+G(d,p) basis set for non-metal atoms [58]. The results reveal the Sharpless route to be the most favored because of its high synchronicity, consistent with other findings that the metal–ligand bonds in vanadium complexes with N and O ligands are easily cleaved in the presence of H_2_O_2_. The Mimoun epoxidation is less favored than the Sharpless mechanism due to its high activation barrier. But it also shows that ligand charge and chosen metal do not represent relevant reasons for favoring this type of mechanism, as revealed in kinetic free energy calculations. The radical mechanism pathway is also highly energy dependent, depicting a high reliance on the electronic distribution in the catalyst structure. It is the various equilibrium changes with active and inactive compounds that eventually lead to the adjustment of the desired epoxidation mechanism.

Another comparative theoretical study on the three mechanisms in olefin substrates catalyzed by vanadium–salen complexes was conducted by Kuznetsov [61], using the DFT level of theory structure geometry optimization with B3LYP (using a (8s7p6d1f)/[6s5p3d1f] basis set for vanadium and the 6-31G(d) for other atoms). The activation threshold depends on the proton’s catalyst location, when one of the oxygen atoms of the salen complex is protonated and the vanadium atom is penta-coordinated with the peroxo-ligand. The protonation of the oxoligand results in an increase in the activation energy by several kJ·mol^−1^. These results are consistent with findings by Shul’pin [62,63], reporting that metal–ligand bonds with N and O are cleaved easily in the presence of H_2_O_2_. This suggests that the intramolecular hydrogen bonding of the amino group is not essential for the catalysis to occur. These calculations also show that the Mimoun mechanism of epoxidation is not favorable due to its high first-stage activation energy barrier, leading to the formation of acetaldehyde instead of the epoxide. Interestingly, the energy consumption for proton migration in the oxygen transfer towards the epoxide displays an intermediate activation barrier between Mimoun and Sharpless routes. With this, several reports stress the Sharpless epoxidation as the tendentially favored mechanism [45].

## 3. Development of Epoxidation Reaction Conditions

### 3.1. Current Overview

**Figure 10:**
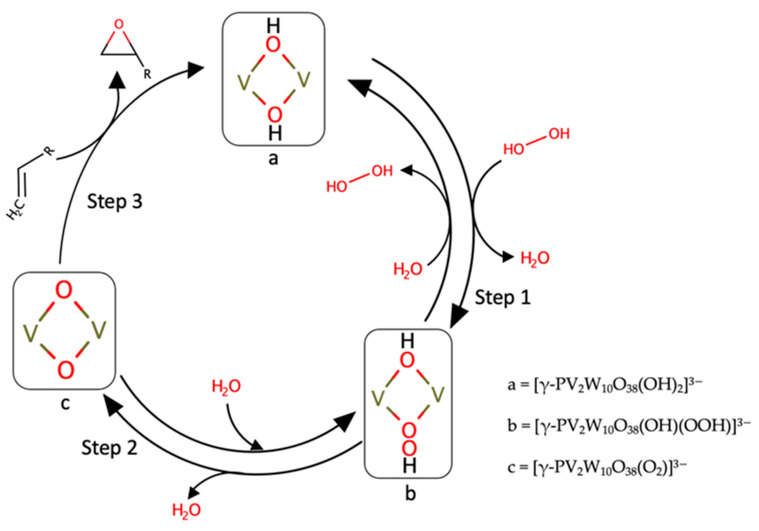
Schematic representation of the mechanism of epoxidation of alkenes with H_2_O_2_ catalyzed by a divanadium-substituted phosphotungstate [65].

Most reported works on ESAs were developed with oxygen-based oxidants with vanadium catalytic systems. Prominently, polyoxometalates such as [VO(acac)_2_] can be introduced in an epoxidation mechanism similar to the ones described above, despite some differences in reaction kinetics (namely, the determining step’s variation in the rate law) [64,65]. We now discuss the importance of kinetic and computational insights in the epoxidation of small alkenes and allylic alcohols with vanadium complexes (with different oxidizing agents), on asserting optimal reaction mechanisms (Figure 10).

An example of small alkene epoxidation is materialized in Figure 10, where we depict the mechanism of epoxidation with H_2_O_2_ catalyzed by a divanadium-substituted phosphotungstate. We can see three main steps described by this epoxidation, yielding a turnover number of approximately 210 [65]. In step 1, the catalyst’s reaction (a divanadium-substituted phosphotungstate) with hydrogen peroxide leads to reversible formation of a hydroperoxo species. In step 2, the successive dehydration of the formed complex into an active oxygen species with a peroxo group occurs. And in the final third step, the complex reacts with the alkene to form the corresponding epoxide. This process can rapidly catalyze this epoxidation (around 10 min), under stoichiometric conditions. The onset development of this method is reported with catalytic trials of different substates, modifications and terminal oxidants, which highlighted several interactions occurring in the reaction’s chemical space [58,66]. This outtake is consistent with the computational studies reported by Aschi using DFT [66] trials. The theory level choice represents a crucial step for obtaining reliable results, given the complex nature of vanadium lability. Also, concerning simulations of different solvents, the structural geometries were optimized with B3LYP [67].

By optimizing molecule geometries, it was stressed that the greatest observed event in the catalytic oxidation of olefins by oxo-vanadium complexes is the formation of aldehyde/ketone derivatives (instead of the corresponding epoxides). These findings fit well with previous experimental results with oxovanadium-based epoxidations of aromatic olefins (like styrene and its derivatives), using H_2_O_2_ as the primary oxidant [68]. They provide key arguments on the reaction mechanism’s activity in the catalytic oxidation of olefins, promoted by the [VO(acac)_2_]/H_2_O_2_ system.

In the Sharpless mechanism, a vanadium-catalyzed asymmetric epoxidation is described through reaction activities and stereoselectivities [69], highlighting one-pot strategies with [VO(acac)_2_]. From employing chiral vanadium catalysts containing bishydroxamic acid ligands, to the successful in situ application of vanadium complexes with the same ligand, previous works reported the epoxidation of tryptophols up to 89% yield through epoxide opening to form hydroxyfuroindoline derivatives [70]. The subsequently reported benefits from electronic distribution and electron-donating within the indole substrate have deeply improved reaction yields.

Major breakthroughs in vanadium-catalyzed methods on allylic alcohol epoxidation came with the use of vanadium-binaphthylbishydroxamic acid (BBHA) and alkenol reactions [71,72,73]. Notably, Noji [71] reported a mechanism using tert-butyl hydroperoxide (TBHP) as an oxidizing agent in toluene. In this work, the optically active BBHA ligands were synthesized from (S)-1,1′-binaphthyl-2,2′-dicarboxylic acid and N-substituted-O-trimethylsilyl (TMS)-protected hydroxylamines via a one-pot, three-step procedure. These epoxidations of substituted allylic alcohols using a vanadium complex were successfully performed in toluene with a TBHP water solution, producing epoxides with 18% to 95% yield (with better enantioselectivity for trisubstituted allylic alcohols), in a minimum of 1 day.

On the other hand, vanadium-salen catalysts (or similar bidentate Schiff base ligands) were also tested with vanadium compounds on alkene epoxidation catalysis. Using TBHP or H_2_O_2_ as oxidants, Grivani [74,75,76] reports the use of precatalytic structures with Schiff base molecules, as solvent polarization decreases the reaction activity. We highlight examples where different solvents, oxidizing agents and catalyst concentrations were studied for epoxidations using vanadium-salen catalysts [74,75,76]. The effect of catalyst concentration in the epoxidation of cycloalkenes, with CCl_4_ as solvent and in the presence of TBHP as oxidant, revealed that increasing the catalyst amount to 1:4 substrate:oxidant ratio resulted in a decrease in epoxidation yield. The 1:3 proportion of cycloalkene:TBHP was considered the best one due to the higher conversion ratio, close to 90% yield in 270 min of reaction time. In this sense, the formation of peroxy-vanadium(V) species in the epoxidation mechanism showed that only the reactions using CHCl_3_ and CCl_4_ as solvents provided viable conversion rates. This is also reported with vanadium(V) complexes [77,78] including a tridentate ONO aroylhydrazone ligand using a solvent mixture of CH_3_OH/CH_2_Cl_2_. With solvent effect studies, the best epoxidation results were found by Grivani with CHCl_3_ as solvent and TBHP as an oxidizing agent, presenting a conversion ratio of 84%. It is also stated that the solvent’s coordinating ability improved overall bonding to the vanadium center, resulting in lower conversion yields. Based on the high epoxidation yield with CCl_4_ (69%) and CHCl_3_ (84%), Grivani [74] stated that both are suitable solvents for radical routes in oxovanadium-catalyzed epoxidation mechanisms. Other vanadium complexes with alternate two bidentate oxygen donor ligands, similar to the Schiff base described above [79,80], also display an excellent performance on olefin epoxidation while using hydrogen peroxide as the oxidant, up to 74% selectivity under mild reaction conditions (4 h at 303 K).

Having described the main developments in vanadium-catalyzed ESAs, we turn to specific examples of tunable epoxidations, concerning sustainable research development.

#### 3.1.1. Vanadium-Catalyzed Epoxidation of Allylic Alcohols

The epoxidation routes of allylic (and homoallylic) alcohols with vanadium-based catalysts are thoroughly explored in the literature. However, given the lower reaction molar ratios between the epoxy alcohols and vanadium complexes (close to 1:1), an outline of the catalytic role of vanadium is yet to be studied. One may argue that such close ratios make these compounds as activators, in accordance with the IUPAC Gold Book [81]. But by far, oxo-vanadium based complexes (mainly [VO(acac)_2_] and [VO(O*i*Pr)_3_]) are found to be the most reported catalysts in harnessing tunable reaction mechanisms for cost-effective epoxidations. We now present some pivotal works on sustainable reaction conditions for several epoxidations of allylic alcohols divided into three major routes: (i) tunable oxo-vanadium complexes; (ii) epoxidation with support elements; and (iii) asymmetric and domino epoxidations.

- Example 1: Tunable oxo-vanadium complexes

This example highlights how common oxo-vanadium structures can be introduced to tunable reaction mediums to improve allylic alcohol epoxidation. The most representative catalyst structures are [VO(acac)_2_] and [VO(O*i*Pr)_3_], as in the presence of an oxidizing agent they induce the epoxidation of homoallylic alcohols. Through variable reaction temperatures and environment settings, Lattazani reported a best enantioselective score of 64% in a 5 h reaction at 40 °C, using a substrate/oxidant ratio ([VO(acac)_2_]/TBHP) of 1.5 mol%/1.3 eq in dichloromethane [82]. Ranging from extreme conditions, such as under an inert atmosphere at −5 °C [83], major strategies with [VO(acac)_2_] and [VO(O*i*Pr)_3_] involve pre-reaction conditions prior to epoxidation. For example, introducing chiral hydroxamic acids in the pre-reaction of oxo-vanadium complexes results in the formation of vanadium-based species capable of improving the catalytic epoxidation of allylic and homoallylic alcohols. By refining good yields and overall enantioselectivity (averaging higher than 90%), they turn oxo-vanadium structures into versatile species, performing catalytic reactions in non-aqueous solvents [55,84,85,86], water [87,88] and active peroxides with achiral hydroxamic ligands [89,90]. Given this adaptable framework, it was even possible to prevent these complexes’ drawbacks in the oxidation of some allylic alcohols to their corresponding ketones and aldehydes through the selective introduction of hydroxamic acids [91]. But, beyond the versatility of hydroxamic acids for cost-effective purposes [92], it is worth mentioning the common coordination issues with oxo-vanadium complexes that may significantly limit epoxidation selectivity for the desired reaction.

Another interesting example for the epoxidation of allylic alcohols with a tunable catalyst is presented as a homogenous catalytic mechanism with pyrone-based oxo–vanadium complexes [93]. Branded as the new generation of catalysts for allylic alcohol epoxidation, the overall performance of these molecules diminishes with ligand complexity, as reaction times vary from 1 h to 9 h in the example of gerianol epoxidation. By presenting an overall epoxidation efficiency comparable with [VO(acac)_2_] complexes (close to 100%), it has the benefit of only using TBHP as the oxidant without needing complex pre-reaction conditions. However, reaction temperatures often climb to high values while organic solvents (such as dichloromethane) are used in parallel, with approximately 1.00 mmol of substrate, 0.02 mmol of catalyst complex and 1.50 mmol of TBHP [93,94]. They are shown to be successful catalyst precursors of allylic alcohols, as Pereira explains that they display a high selectivity conversion rate, comparable to oxovanadium-based catalysts [93]. Given the immobilization strategies for overcoming the coordination drawbacks of the [VO(acac)_2_] heterogeneous catalytic systems, these complexes present as an ideal alternative scaffold for sustainable alternative designs.

- Example 2: Epoxidation with support elements

**Figure 11:**
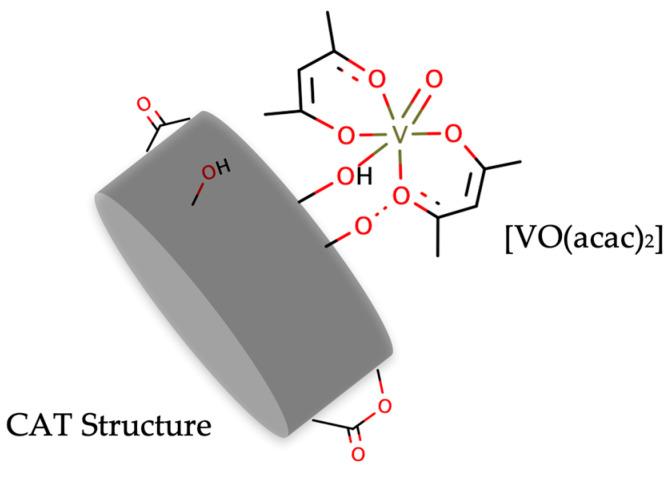
Schematic representation of [VO(acac)_2_] catalyst inserted through the oxygen subunits of CAT1 [95].

Considering the disadvantages mentioned in the previous example, new strategies in heterogeneous catalysis systems have been developed to overcome structural and reaction constraints. An interesting take is the deployment of nanostructured materials as catalyst supports for oxo-vanadium catalysts. The idea is to use external support elements to overcome the catalyst immobilization issues described in other reaction examples. For example, a [VO(acac)_2_] precursor using TBHP was inserted through the oxygen subunits of carbon (CAT) layers with amine groups as support elements (Figure 11). Results showed that the resulting materials can yield epoxide conversions of around 90% with a close to 99% selectivity [95].

For the epoxidation of geraniol (1.0 mmol), 0.10 g of the heterogeneous catalyst was mixed in a dichloromethane medium at room temperature with continuous stirring, as 1.5 mmol of the oxidant TBHP was progressively added [95]. In this example, CAT subunits display high catalytic performance with conversions around 90% to 100%, displaying smaller reaction times (2.5 h vs. 48 h), of other heterogeneous [VO(acac)_2_]-based catalysts. This is explained as a consequence of the immobilization of the vanadium complex at the surface of the CAT rods, easing access of the reactants to the catalytic center. This in turn improves mass transport and catalytic reaction rate for optimal performance. The outlined route is an important example of the development of efficient bulk heterogeneous [VO(acac)_2_] systems with negligible diffusion limitations.

Parallel to the aforementioned process, another example of heterogenous catalytic complexes is the use of catalyst structure ligands. Studies reported the use of [VO(acac)_2_] complexes with amine-activated carbons using TBHP as the oxidant with high catalytic activity (a 93% conversion ratio and a mmol ratio of converted epoxide:vanadium complex/reaction time of 0.6 h^−1^) in dicholomethane at 0 °C [96]. In a recent work, *α*-amino acid anions serve as chemical ligands to small nanostructures, in an assembly of rigid inorganic layers around the chiral center [97]. This disposition restricts the chemical space by guiding the trajectory of the remaining reactant molecules to a stable and firm environment. Different trials revealed reaction yields from 50% to 90%, but there is a reported advantage in catalyst recycling in the epoxidation of selected alcohol. In recycle runs for the epoxidation of 2-methyl cinnamyl alcohol with water-soluble VOSO_4_ as the source of the catalytic center, epoxide yields keep well above 90% after three consecutive runs (up to 1440 min) [96]. While preserving overall enantioselectivity, it has been reported that the nature of multiple interactions in the vanadium/nanosheet *α*-amino acid systems reveals excellent reusability of these heterogeneous catalysts. However, they depend on the trade-off between the existing non-covalent interactions with the complex and its layers. By restricting solid/liquid interfaces, the catalytic epoxidation occurs under “pseudo-homogeneous conditions”, which can increase the overall yield (in one case, from 83% to 93%) in half the time (1050 to 520 min). After further validation, confirming the chiral induction from the amino acid sheets, other types of nanosheets can be considered for various types of ligands and catalysts. This environmentally friendly take is further emphasized when, in subsequent trials using water as the solvent, the catalyst can be separated from the epoxidation products through liquid/liquid extraction, easing the catalyst recycling process. As such, these planar substituents enhance the enantioselectivity of allylic alcohol epoxidation (in some cases by 40%) through chemical synergy and hydrogen bonding mechanisms [98,99], in a different route from the previously described homogeneous processes.

Other strategies were reported for fine-tuning catalytic enantioselectivities in the vanadium-based epoxidation of allylic alcohols [100]. Zhao presented a ligand-design strategy that also employs inorganic nanosheets for modifying *α*-amino acids as chiral ligands for active vanadium centers. In the epoxidation of 2-(2,2-dimethypropyl)allylic alcohol, known for its issues in the substrate accessing the vanadium center, an improved yield of the isolated epoxy alcohol of 95% was observed. Different trials show the enantioselectivity between 33% and 53% over 6 h of reaction time, as the enantiomeric excess was 33% to 39 %. This increase in enantioselectivity is due to the LDH layers, brought as catalyst ligands. While these are interesting advantages, the use of epoxidation fine-tuning with structural support reveals that bulkier substrates have increasing difficulty in diffusing into the metallic catalytic center. This is backed by the recent work of Cohen [101], where heavy alkenyl cyclopropyl carbinol substrates were only epoxidized with [VO(acac)_2_] complexes due to the impressive selectivity from the rigid substrate’s cyclopropyl core. With more than 75% yield and a product ratio of 98:02, the proposed layer-models had no impact when they were exposed to the catalytic sites, as an improvement in enantioselectivity was achieved (explained due to the steric effects of the epoxidation). As such, the proposed methodology must consider the type of substrate for further development and scaling up for catalytic epoxidation design.

- Example 3: Asymmetric and domino epoxidations

**Figure 12:**
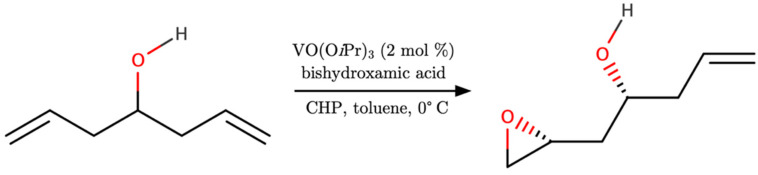
Schematic representation of enantioselective asymmetric epoxidation of a *meso* secondary homoallylic alcohol with [VO(O*i*Pr)_3_] as catalyst [55].

In this final example, we report on two particular types of fine-tuning epoxidation of allylic alcohols. The first one is perhaps one of the most successful cases: the low-cost process of producing florfenicol via a vanadium-catalyzed asymmetric epoxidation [80]. It is characterized by using readily available materials with mild reaction conditions and a convenient catalytic work-up. Using [VO(O*i*Pr)_3_] as the catalyst, an enantioselective reaction with roughly 37% yield of florfenicol was achieved with bis(hydroxamate) compounds (Figure 12), using available methylthiobenzaldehyde derivatives. The limitations of the large-scale preparation of florfenicol are known due to the unavailability of (R)-hydroxynitrile lyase as a biocatalyst with subsequent unsatisfactory yield.

To improve enantioselectivity in asymmetric epoxidations, reaction conditions (catalyst, solvent, temperature and catalyst loading) were thoroughly screened in other studies [55]. Focusing on a low-cost procedure, the ideal setting revealed the use of CHP as oxidant and toluene as solvent, at 0 °C (Figure 12). The best reactions produced yields up to 56%, over 8 days of reaction time, with an enantiomeric excess of 94%. But several drawbacks were reported as an increase in reaction temperature led to a sharp decrease in enantioselectivity, with no significant improvement through increased catalyst loading.

Besides homo and heterogenous strategies with vanadium-based catalysts, the second particular example we highlight are epoxidation domino (or cascade) reactions. This process is described as two or more chemical transformations occur on the reaction step, with no additional additives or condition changes [102,103]. They are employed when sustainable chemical process design is prioritized, using one-pot strategies to reduce time and resource consumption. This is especially critical in reactions involving natural and fossil-based raw materials [104,105,106,107] as they have proven results in reaction stereo-control while needing mild reaction conditions. Some works report an efficient enantioselective chiral vanadium-catalyzed domino epoxidation with a robust library of tryptophols [70]. This enantioselective epoxidation reaction with ring-opening cascades, using [VO(acac)_2_] complexes, produced modest overall yields (up to 62%) while displaying high enantio-selectivity results (82%) at 0 °C in 12 h reaction time. Due to the inherent stability of the chiral vanadium catalyst in relation to moisture and air, these reactions can be air operated with a readily available aqueous oxidant (70% weight aqueous solution of TBHP for the present reaction), using toluene as solvent at low temperatures. The catalyzed dearomatization and intramolecular epoxide ring-opening cascade was stated to develop polycyclic compounds, but it revealed that small substrates are the ideal candidates for this strategy, given their tunable conditions [70].

#### 3.1.2. Vanadium-Catalyzed Epoxidation of Alkenes (Olefins)

As with the previous section, we present current works on the assessment of different sustainable epoxidations conditions, now considering small alkenes as substrates. The chemical epoxidation of alkenes and olefin-based compounds have been developed as an engineered response to yield industry-valuable chemicals. Several oxidizing catalysts have been targeted, specifically atomic oxygen and liquid peracids, while vanadium Schiff base compounds were regarded as high performing catalysts for high-impact epoxidation. The following examples regard these major characteristics, targeting potential catalytic activity and selectivity [108].

- Example 1: Outer and inner sphere epoxidations

**Figure 13:**
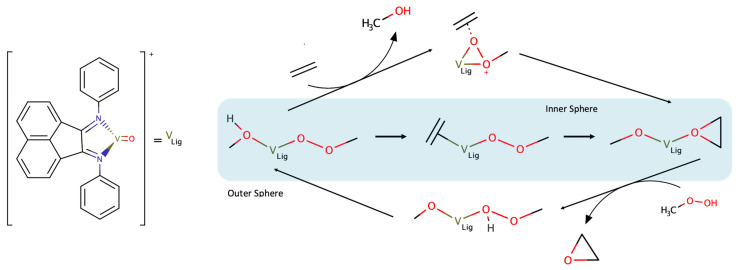
Epoxidation reaction scheme for oxovanadium-based complexes with olefins [29].

A well-known example of small alkene epoxidation using H_2_O_2_ as oxidant (or TBHP in some cases), is the simultaneous occurrence of Sharpless- (outer sphere) and Mimoun-type (inner sphere) mechanisms. Backed by experimental and computational studies (Figure 13) it constitutes an interesting advantage when harnessing lability for overlapping chemical benefits from both strategies at a reduced cost. A recent study on mechanistic insights of this example is provided by Freindorf [109], claiming that after the activation of the vanadium(IV) complex, these two competitive processes may occur, corroborating previous theoretical explanations by Calhorda [29]. While one path is considered an “outer sphere path” with an external attack of the olefin at the coordinated peroxide, the other, an “inner sphere mechanism”, is based on the vanadium catalyst complex with a coordinated substrate.

Variations in catalytic activity can be explained by an exchange reaction, representing a cycle closing through the replacement of methanol by another substrate molecule. These are endergonic processes as the equilibrium is shifted backwards by increasing alcohol concentrations (as it is a reaction by-product). Reaction turnovers as high as 90% were reported after a reaction time of 24 h at 328 K, with a molar ratio of substrate:oxidant:catalyst of 100:200:1 [29]. The main sustainable concerns rely on solvents with the use of dichloromethane with TBHP as oxidant and acetonitrile with H_2_O_2_. However, as both strategies yield high selectivity towards epoxide formation, this puts them in consideration for optimization strategies for the selective epoxidation of enantiopure olefins and bio-waste raw materials.

- Example 2: Epoxidation with Schiff base ligands

The vanadium complexes presenting tetradentate Schiff base ligands have been used as catalysts for high-impact epoxidation of alkenes. In the example of cyclohexene [110], notably represented with the works of Mohebbi and Boghaei [47,108], its vanadium-catalyzed aerobic oxidation proceeds with a high selectivity at 79–81 °C (up to 61% with oxovanadium complexes). In the presence of a flow of O_2_ for 24 h, using vanadyl catalysts in acetonitrile or DMF as solvent, cyclohexene is oxidized as vanadium(V) can selectively interact with cyclohexenyl-hydroperoxide and cyclohexene to yield an epoxide and an allyl alcohol. However, hydroperoxide heterolysis probably proceeds via an inner sphere process, requiring peroxide coordination to the metallic center. Then, the nucleophilic attack of a non-coordinated olefin on the electrophilic peroxide oxygen atom bound to the metal yields the final epoxide. For cyclohexene epoxidation with an oxovanadium Schiff base complex, Dekar [111] reports 86% epoxide turnout using chloroform as the solvent, with 4μmol of oxovanadium complex, 1 mmol cyclohexene and 3 mmol H_2_O_2_ as oxidant, during 5 h at 80 °C. While the use of chloroform might be avoidable, using H_2_O_2_ encourages a sustainable approach, given the previously reported epoxide oxidants.

Mohebbi and Boghaei also show that the correlation between epoxidation potential and activity seems unclear concerning structural effects. This may deepen the possibilities for reaction manipulation reactivity as both electronic and steric factors can be independently and simultaneously controlled. Ligand substitution distortions may influence energy reorganization towards the transition state in the metallic center in a redox reaction, thus lowering the overall activation energy (relatively Schiff base to the less distributed salen system). These insights are particularly interesting towards olefin epoxidation catalysis, as it has been revealed that performance can be raised by diminishing the number of electric donors’ ligands, while improving epoxide selectivity (up to 80%) with increasing catalyst presence [108].

**Figure 14:**
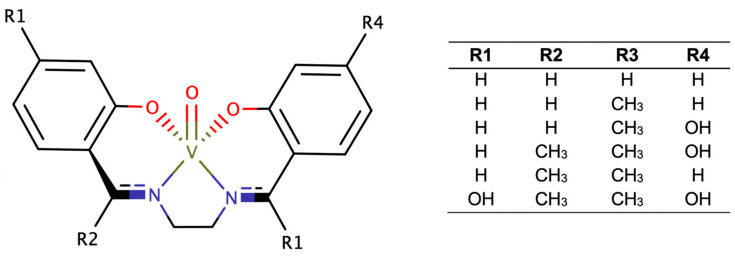
Structure of oxovanadium(IV) complexes used by Mohebbi [48].

Other works on oxovanadium(IV) molecules containing N_2_O_2_-donor ligands were developed by Mohebbi [48] with the purpose of yielding epoxides in a more sustainable way. Reaction yields up to 60% were reported using these ligands in the epoxidation of small cycloalkenes with a blended polar organic solvent (mainly acetonitrile:DMF in a 3:2 ratio) in a 24 h period with O_2_ [48]. This study is particularly relevant due to its mechanistic insights into catalyst modulation by ligand substitution control. It goes as far as reporting equal performance similar to current olefin epoxidation routes (e.g., cyclohexene) with regular epoxidation using vanadium-based catalysts. However, there is a structural difference: a steric effect on the reaction product was verified, causing a cyclic distortion in the produced epoxide which can cause lower epoxidation yields. Despite this, Mohebbi postulates that the redox process with the vanadium Schiff base is fully reversible, revealing that vanadium(V) geometry is stable in both oxidation states. In the specific case of the epoxidation of cyclooctene, the vanadyl complexes decompose to organic intermediate peroxides via a heterolytic mechanism with increased epoxide yield. It shows that catalytic activity improved with a decreasing number of electron-donating groups, as the vanadyl-catalytic complexes are more stable during the oxidation process. Although this is mostly due to its stronger donor ligands, its bulkiness may strongly influence overall activation energy in comparison to the less distributed vanadium(salen) complexes (Figure 14). To investigate this, vanadium-based mononuclear complexes [112] were capable of epoxidizing cyclooctene with 95% yield after testing various reactions conditions (with water, acetone, chloroform or a solvent-free atmosphere), concluding that best results were achieved with acetonitrile as solvent at 70 °C for 2 h). Such a situation provides an opportunity for connecting the results of basic research to develop practical catalysts. The catalytic system described is an efficient method for the epoxidation of cyclooctene, with the advantages of high activity, selectivity and a short reaction time. A recent study by McCaffrey [113] also shows the selectivity of vanadium(V) complex geometry towards epoxidation given the chosen ligand and reaction oxidant. Product turn-out is favored with a methoxy complex, using hydrogen peroxide as solvent, highlighting preferential conditions for using vanadium(V) in small alkene epoxidation.

Other examples show that chemical complexes of oxovanadium-bearing tetradentate Schiff base ligands can also behave as catalysts in the oxidation of styrene and cyclooctene using the same oxidant [114]. Cyclooctene was epoxidized with almost 100% conversion with a molar ratio of 3:1 of TBHP:cyclooctene (as oxidant and substrate) in chloroform at 61 °C over 6 h. Other reported cases highlight the versatility of tridentate Schiff base ligands, catalyzed by an acetophenone radical in an organic solvent with TBHP, bearing a highly selective epoxidation of cyclooctene [115,116]. This complex can generate cyclooctene epoxide yields of 95% with 100% selectivity, using 10 mmol of substrate, 0.032 mmol of catalyst and 30 mmol of TBHP during 4 h in chloroform at 61 °C.

- Example 3: Tunable vanadium complexes

**Figure 15:**
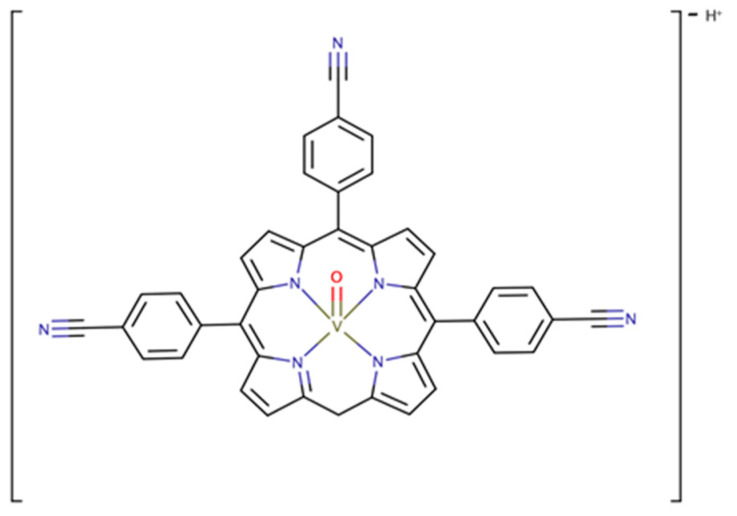
Representation of oxo(peroxo)(corrolato)vanadium(V) for epoxidation of cycloalkenes using KHCO_3_ as a promoter [117].

In this final example, we present different approaches for alkene epoxidation considering a variety of vanadium-based catalytic scaffolds. This selection gathers more examples than regular-based catalysts such as [VO(acac)_2_] when considering the same chemical conditions. Nayak [117] provides an interesting approach by reporting oxo(peroxo)(corrolato)vanadium(V) as a catalytic species for the epoxidation of several cycloalkenes, using H_2_O_2_ as the oxidant and KHCO_3_ as a promoter (Figure 15). This was possible by harnessing an electrophilic addition reaction medium, making unusual corrole-based ligand systems a viable option for sustainable epoxide production. With 1 µmol of oxo-vanadium(IV)-corrolato complex as catalyst, 5 mmol of styrene yielded 50% of the corresponding epoxide, in a acetonitrile/water medium, with 15 mmol of H_2_O_2_ during 1 h of reaction time. Another similar example is the electrolythic aerobic epoxidation of cyclohexene at 70 °C, which is mainly based on [VO(acac)_2_] catalyst quantity screening [118]. Aside from catalyst pre-preparation reactions, this route poses a credible alternative by not compromising on using complex polar solvents or other costly reaction mediums.

Other examples in alkene epoxidation gathered different chemical kinetic data to back up vanadate-imidazole-catalyzed epoxidations, using hydrogen peroxide as the oxidizing agent [41]. As it appears that the rate-determining step is the exchange of oxygen molecules from the catalyst to the substrate, this is well illustrated in the study of tripeptide glutathione with a vanadium-based complex as catalysts for cyclohexene epoxidation. Here, the use of toluene at 80 °C drives the generation of both epoxidation and hydroxylation products, reporting only a 12.4% epoxide yield in a 90 min reaction time [119]. Kinetic data also support the use of vanadium(IV) compounds, as they show higher yields of cyclohexene epoxidation when associated with other transition-metal complexes. Vanadium(IV) complexes from salicylaldehyde scaffolds and chiral diamines also mainly act as catalysts for the epoxidation of styrene (71% conversion rate) and cyclohexene (66% conversion rate) [120]. Optimal reaction conditions report the use of 5 mmol of substrate for 0.1 mmol of catalyst, 30% aqueous H_2_O_2_ as oxidant in acetonitrile at 80 °C. A recent development considering solvent sustainability on styrene epoxidation reported 99% epoxidation selectivity with 97% conversion ratio with dioxvanadium(V) complexes without using organic solvents [121]. In a two-step process through a peroxovanadium intermediate that boosts a renewable catalytic cycle, the catalyst complex substituted with N,N-donor ligands and bipyridine secured overall selectivity to start the epoxidation without an organic solvent. The best reaction conditions were observed using a molar ration of 1:50 of catalyst:styrene with 30% H_2_O_2_ (20 mmol), at room temperature during 8 h of reaction time.

**Figure 16:**
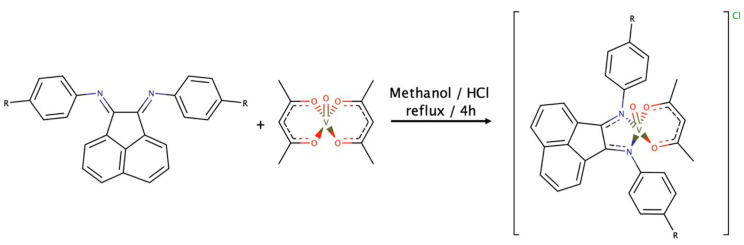
Schematic representation of (pre-reaction) synthesis example of [VO(acac)_2_]-based active catalysts [29].

Also similar to the pre-reaction examples reported on the epoxidation of allylic alcohols, oxovanadium(salen) and [VO(acac)_2_] catalyze the epoxidation of isosafrole with TBHP or hydrogen peroxide as oxidants. Comparing these two catalyst systems, the oxovanadium(salen) complex yielded the best epoxide/by-product ratio of 51%, using TBHP as oxidant and acetonitrile as solvent (in presence of acetic acid) [49]. The oxovanadium complexes with chelating ligands within the equimolar proportion show catalytic properties towards the epoxidation of styrene, cyclohexene and trans-stilbene in acetonitrile [50]. In an essential work by Calhorda [29], mononuclear oxovanadium(IV) complexes were developed and tested for their catalytic activity in the epoxidation of various olefins, using TBHP or H_2_O_2_ as oxidants (similar to the above-mentioned outer and inner sphere epoxidations). Kinetic and DFT studies were performed, naming the nature of the catalytic species and reaction mechanisms. Through a pre-reaction shown in Figure 16, the stability of VO(IV) complexes towards oxidation was tested, as they may be easily oxidized to vanadium(V) during epoxidation. This is an important issue for understanding how the mechanisms of catalytic epoxidations are promoted by oxovanadium(IV) complexes and the nature of the active catalytic species (in particular, the vanadium’s oxidation state).

The presented oxovanadium(IV) complexes cited by Calhorda were found to be active catalyst precursors for the epoxidation of small olefins, using either TBHP or H_2_O_2_ as oxidants. All of them presented a high selectivity toward epoxide formation as no diol formation was observed. Still, other reaction mechanisms and pre-catalyst routes are rarely explored given the complexity of these mechanisms, in a similar aspect to the epoxidation of allylic alcohols.

Bimetallic vanadium-based catalysts were also explored for olefin epoxidation. Displaying comparable reactivity for such epoxidation route, the electronic influence of the ligand towards the metallic center does not seem to change from a single-metal atom complex. This is reported with molecules where the metallic centers are secluded from each other, presenting either nitrogen or oxygen atoms at the binding sites [122,123]. Styrene (1 mmol) was reported to be epoxidized using a vanadium BOX complex (0.026 mmol) in acetonitrile, with a 91% conversion rate and 45% epoxide yield, using TBHP (2 mmol) as oxidant in a 6 h reaction time [123]. These findings can be explained as bimetallic vanadium catalysts (displaying one V-V metallic bond) can also be split in separate fragments when they are bridged with an oxide bond. Thus, they each display similar catalytic activity for homogenous olefin epoxidation, limited from using hydrogen peroxide as oxidant, as seldomly reported in heterogeneous vanadium catalysts [124,125,126]. In a representative study of cyclooctene epoxidation, Agustin reported a high turnover frequency of 2409 h^−1^ (cyclooctene transformed:catalyst:time at 20 min) using a solvent-free medium at 80 °C with a substrate:complex molar ratio of 2000:1 (and 32% selectivity) [124]. It highlights the consistent development of sustainable reaction conditions, by using TBHP as oxidant in a solvent-free reaction medium.

### 3.2. Overview on Sustainable Processes

With these examples, we have covered ESAs’ development with vanadium-based catalysts towards sustainable processes. Despite the epoxidation routes’ complexity here presented and the unfolded mechanisms still to be fully understood, many outtakes on sustainable epoxidation can be provided. In both alkenes and allylic alcohols, we see a predominant choice of vanadium-based ligands, mainly [VO(acac)_2_], [VO(O*i*Pr)_3_] and Schiff base ligands, with H_2_O_2_ and TBPH as the primary oxidant choice. Tunable routes can differ in specific types of ligands or external structures that support catalytic epoxidation, which confer different characteristics with different substrates. Although we cannot forward a broad solution for this type of epoxidation, foundational information was gathered to move experimentalists in selecting different types of routes concerning laboratory and industrial constraints.

Addressing the established concern of circular economy-friendly development strategies, we have reported recent advances in the synthesis of new catalytic complexes to epoxidize simple molecules obtainable from bio-waste recovery, like cyclohexene, styrene or limonene. In this spectrum [29], [VO(acac)_2_] complexes using H_2_O_2_ and TBHP as oxidants can yield as high as 90% efficiency with different choices of solvents. Aside from bidentate ligands in vanadium-oxidized species, catalysts with tridentate and tetradentate ligands were also reported as good catalytic alternatives [59,75,127,128,129,130,131].

Regarding issues in catalyst loading and reaction environments, in both cases of ESAs, a reported trade-off between ligand lability and epoxidation activity was often reported. To this end, vanadium based-catalysts display interesting performances in forming active vanadium-peroxo complexes [132], as one hypothesis comes from the influence of ligand electronics during the catalytic process (in complexes with nitrogen or oxygen donors) and assessing catalyst loading [61]. As bidentate vanadium complexes with increasing solvent polarity diminish epoxidation yields, it gives an explanation for the coordination of polar species (such as TBHP) on the vanadium center, thus decreasing the number of available active vanadium peroxide complexes in solution. Furthermore, these ligands were also shown to epoxidize alkenes and allylic alcohols with greater substrate complexity in the presence of H_2_O_2_. Pereira [93] described variations in the oxo-vanadium complexes, such as pyrone-based, to be the most efficient catalysts exhibiting performances comparable to that of the well-known [VO(acac)_2_] (close to 100% in about two hours of reaction time). The kinetic studies suggest that this occurs due to solvent insolubility, although complete solubilization is experimentally confirmed during the catalytic cycle.

With the reported studies herein, backed by kinetic and computational studies, we have provided an added insight on the dependency of ESAs’ catalytic activity with the nature of the vanadium-based catalysts and ligands, which certainly must be explored in future catalytic research endeavors.

## 4. Final Remarks

This review highlights the growing contribution of the vanadium-based epoxidation catalysis of allylic alcohols and small alkenes towards the development of sustainable reaction mechanisms. The depiction of well-established know-how on vanadium-catalyzed epoxidations, including a relevant number of highly efficient enantioselective reactions, has been demonstrated. But despite these advances, key challenges still persist. The research on chiral vanadium catalysts, with the ability to stereo-selectively oxidize unfunctionalized olefins, presents a remarkable problem. It stressed the need for additional kinetic and mechanistic studies to uncover the pivotal role of chiral vanadium compounds towards efficient and selective sustainable processes.

One of the insights brought by this review is the catalogue of successful methodologies that overcame the trade-off between ligand lability and epoxidation activity using vanadium based-catalysts. The influence of ligand electronics during the catalytic process demonstrates how vanadium complexes can diminish epoxidation yields with increasing solvent polarity, affecting active complexes in solution. The presented reaction mechanisms gave structured concepts for process development, considering the breakdown of the proposed epoxidation mechanisms.

On sustainable development remarks, we have identified the most sought-out variable elements in vanadium-based epoxidation research. For either small alkenes or allylic alcohols, several articles use different catalytic structures as scaffolds with different radicals ([VO(acac)_2_], [VO(O*i*Pr)_3_] and Schiff base ligands) in various reaction mediums (from water to polar organic solvents), with important oxidizing features from H_2_O_2_ to TBPH. While asserting reaction performance, the reported combinations of solvent variability, reaction oxidants and different substrates showed how the design of economically viable routes can be challenging. However, proper development must be supplemented with computational and kinetic studies, further enhancing the predictability of the successful design of novel epoxidation processes.

## Figures and Tables

**Table 1:** Overall number of research papers concerning the terms “epoxidation” and “vanadium catalysts” (Source: Web of Knowledge).

Publication Years	Article Count	Percentage/%
2011–2020	262	53.4
2001–2010	168	33.8
1991–2000	61	12.3
1981–1990	6	1.2

## Data Availability

Data sharing is not applicable to this article.
